# Accuracy and reliability of tridimensional electromagnetic sensor system for elbow ROM measurement

**DOI:** 10.1186/s13018-022-02961-5

**Published:** 2022-01-29

**Authors:** Kohei Yamaura, Yutaka Mifune, Atsuyuki Inui, Hanako Nishimoto, Takeshi Kataoka, Takashi Kurosawa, Shintaro Mukohara, Yuichi Hoshino, Takahiro Niikura, Kouki Nagamune, Ryosuke Kuroda

**Affiliations:** 1 Department of Orthopaedic Surgery, Kobe University Graduate School of Medicine, 7-5-2, Kusunoki-cho, Chuo-ku, Kobe, 650-0017 Japan; 2 Human and Artificial Intelligent Systems, University of Fukui Graduate School of Engineering, Fukui, Japan

**Keywords:** Elbow joint, Electromagnetic sensor system, Range of motion

## Abstract

**Background:**

While the precise measurement of the range of motion (ROM) of the elbow joint is important for clinical assessment and rehabilitation, problems include low accuracy and reproducibility in goniometer measurements due to the influence of soft tissue. The purpose of this study was to validate elbow joint motion analysis using a three-dimensional electromagnetic sensor system (EMS).

**Methods:**

The accuracy and reproducibility of the EMS system were evaluated at four angles (0°, 45°, 90°, and 135°) using a model bone of the humerus and forearm. In addition, the maximum extension and maximum flexion of six elbows of six healthy volunteers were assessed by radiographic and EMS measurements. Accuracy was assessed by calculating the mean value of the measurement angle, standard deviation, Pearson’s correlation coefficient, and the Bland–Altman method. Reproducibility was assessed by calculating the intra-rater and inter-rater reliabilities using intraclass correlation coefficients.

**Results:**

In the model bone evaluation, the mean angles of the EMS measurement were 1.2° ± 2.0°, 45.4° ± 2.1°, 91.7° ± 2.4°, and 134.6° ± 2.7° at 0°, 45°, 90°, and 135°, respectively. In the in vivo evaluation, the elbow angles at the maximum extension with the EMS and radiographic angles were 4.7° ± 3.0° and 2.7° ± 2.0°, respectively, and the angles at maximum flexion were 131.8° ± 13.0° and 130.8° ± 4.5°, respectively. There were statistically significant correlations between the EMS and radiographic measurements; the Bland–Altman plots indicated that the two methods were almost in agreement for both extension and flexion.

**Conclusions:**

This method of measuring ROM of the elbow joint using EMS showed high accuracy, reliability, and reproducibility. The current results demonstrated the possibility of using the electromagnetic system to provide an accurate evaluation of the elbow joint in clinical settings.

## Background

Precise measurement of the range of motion (ROM) of the joint is essential for clinical assessment in outpatient clinics and operating rooms. The universal goniometer (UG) has been widely used to measure ROM of the joint in the clinical setting by many medical practitioners, such as orthopedic surgeons and physical therapists, and is considered the gold standard [1, 2]. Although the UG is a simple measuring tool, there are problems of low accuracy and reproducibility due to the influence of soft tissue [3, 4]. Several studies have reported that UG has fair intra-examiner reliability but poor inter-examiner reliability [1, 5]. Radiographic measurement for ROM evaluation has been proposed as an alternative to UG. This method has shown a higher level of accuracy; however, it requires a radiological imaging system, time, cost, and even radiation exposure for the patient [6].

A quantitative assessment method of knee motion with high reproducibility using a three-dimensional electromagnetic sensor system (EMS) has been recently reported [7–13]. A three-dimensional motion tracking system using electromagnetic sensors has a high accuracy and a high sampling rate when capturing the relative movement between the objects and has been applied for joint motion assessments with the benefit of non-invasiveness to the human body. Although this highly accurate EMS has been frequently applied to the evaluation of knee joint function, there have been no reports of its application to the elbow joint. The elbow joint is a trocho-ginglymoid joint that articulates the distal humerus with the proximal radius and ulna, allowing wide ROM in flexion and extension movements [6]. We hypothesized that EMS could be used to measure the ROM of the elbow joint with high accuracy and reliability. Therefore, the purpose of this study was to validate a new application of elbow joint motion analysis using a three-dimensional EMS.

## Methods

### Experimental setup

The extension and flexion angles of the elbow joints were measured using an electromagnetic device (Liberty®, Polhemus, VT, USA). The system consists of a transmitter that produces an electromagnetic field and three-dimensional electromagnetic sensors. This system had a root-mean-square accuracy of 0.76 mm for position and 0.15° for orientation when it was used within an optimal operational zone with transmitter-to-sensor separation within 106 cm, and there was no interference from magnetic material [14].

**Fig. 1:**
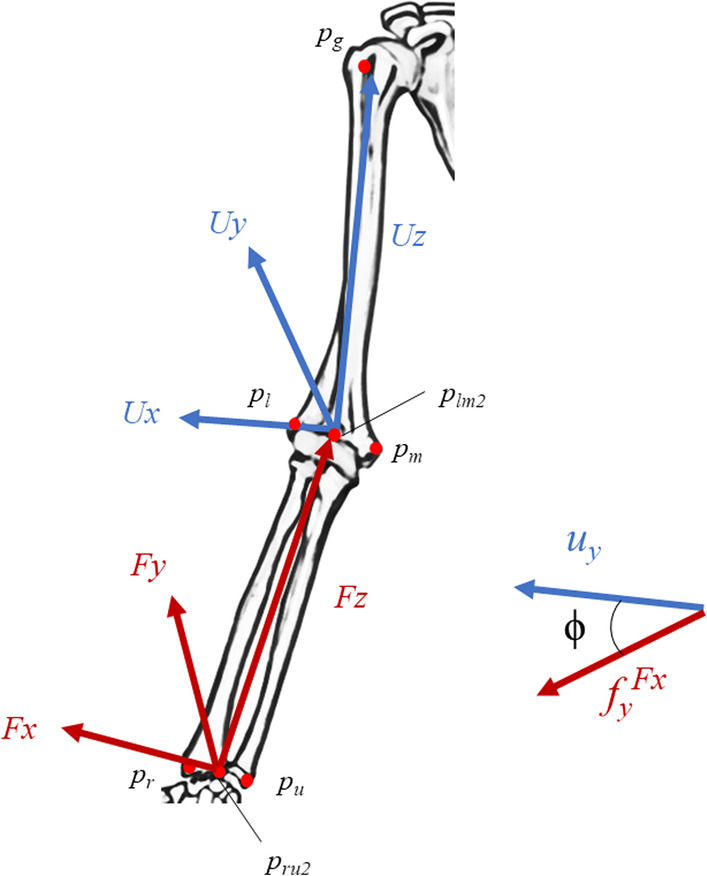
Diagram of the coordinate system. Anatomical landmarks on the humerus, radius and ulna (*p*_g_; greater tubercle of humerus, *p*_m_; medial epicondyle of humerus, *p*_l_: lateral epicondyle of humerus, *p*_r_: styloid process of the radius, *p*_u_: styloid process of ulna, *p*_lm2_: midpoint of *p*_m_ and *p*_l_, and *p*_ru2_; midpoint of *p*_u_ and *p*_r_). The elbow joint flexion angle *ϕ* was defined as acos (*u*_*y*_ · *f*_*y*_)

Two electromagnetic sensors were fixed to the humerus diaphysis and ulna diaphysis. The third electromagnetic sensor was used to register the three-dimensional positions of the five bone-based landmarks (*p*_g_: greater tubercle of humerus, *p*_m_: medial epicondyle of humerus, *p*_l_: lateral epicondyle of humerus, *p*_r_: styloid process of the radius, and *p*_u_: styloid process of ulna) in relation to the other two fixed sensors (Fig. 1). If the midpoints of *p*_m_ and *p*_l_ are *p*_lm2_, the coordinate system of the upper arm is defined by *Uz* = *p*_g_—*p*_lm2_, *Uy* = (*p*_l_—*p*_lm2_) · *Uz*, and *Ux* = *Uy* · *Uz*. If the midpoint of *p*_u_ and *p*_r_ is *p*_ru2_, the coordinate system of the forearm is defined as *Fz* = *p*_lm2_ − *p*_ru2_, *Fy* = (*p*_r_ − *p*_ru2_) − *Fz*, and *Fx* = *Fy* − *Fz*. The three-dimensional position data of the landmarks were converted to set the coordinate system of the elbow joint movement [15]. The elbow joint flexion angle is determined by *ϕ* = acos (*u*_*y*_ · *f*_*y*_) if the unit vector shown in Fig. 1 is used.

### Assessment of EMS accuracy and reproducibility

**Fig. 2:**
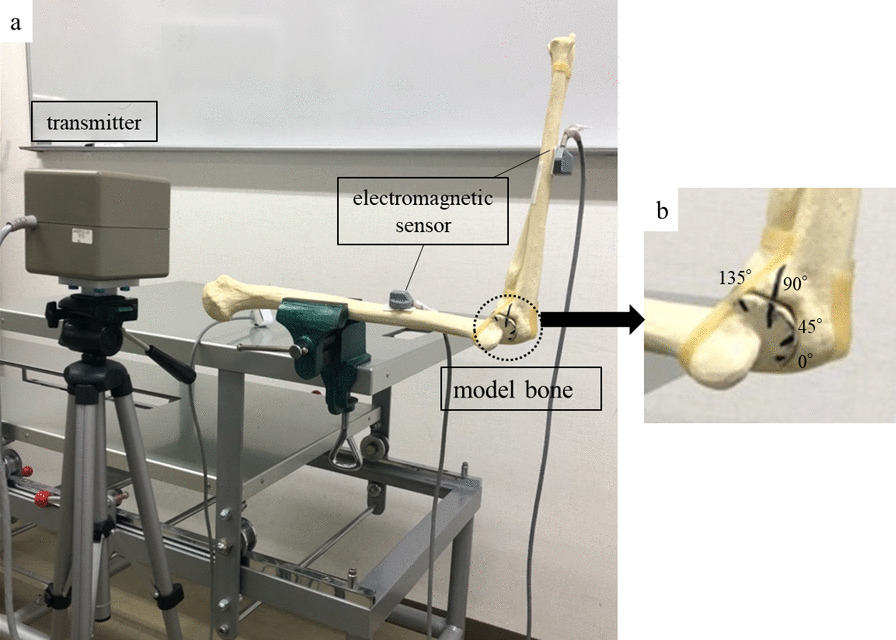
Evaluating the accuracy and repeatability of EMS using model bones. **a** The two sensors were fixed to the humerus and ulna diaphysis. **b** The examiner paused for approximately 3 s at the four actual measurement angles of 0°, 45°, 90°, and 135° of elbow flexion as measured and marked by the goniometer beforehand

The accuracy and reproducibility of the system were evaluated using a model bone of the humerus and forearm (Fig. 2a). The two sensors were fixed to the humerus and ulna diaphysis, as described above. In addition, the humerus was fixed as shown in Fig. 2, and one examiner grasped the forearm and flexed the elbow joint passively from the extended position at 90° of forearm supination. The examiner paused for approximately 3 s at the four actual measurement angles of 0°, 45°, 90°, and 135° of elbow flexion as measured and marked by the goniometer beforehand (Fig. 2b). The angles between the humerus and ulna were analyzed at four angles using a goniometer and electromagnetic system. Measurements were taken ten times each by two experienced hand surgeons. Accuracy was assessed by calculating the error between the mean value of the measurement angle with the EMS and the actual measurement angle, standard deviation (SD), and Pearson’s correlation coefficient. Reproducibility was assessed to calculate intra-rater and inter-rater reliabilities using intraclass correlation coefficients (ICCs).

### In vivo assessment of EMS

**Fig. 3:**
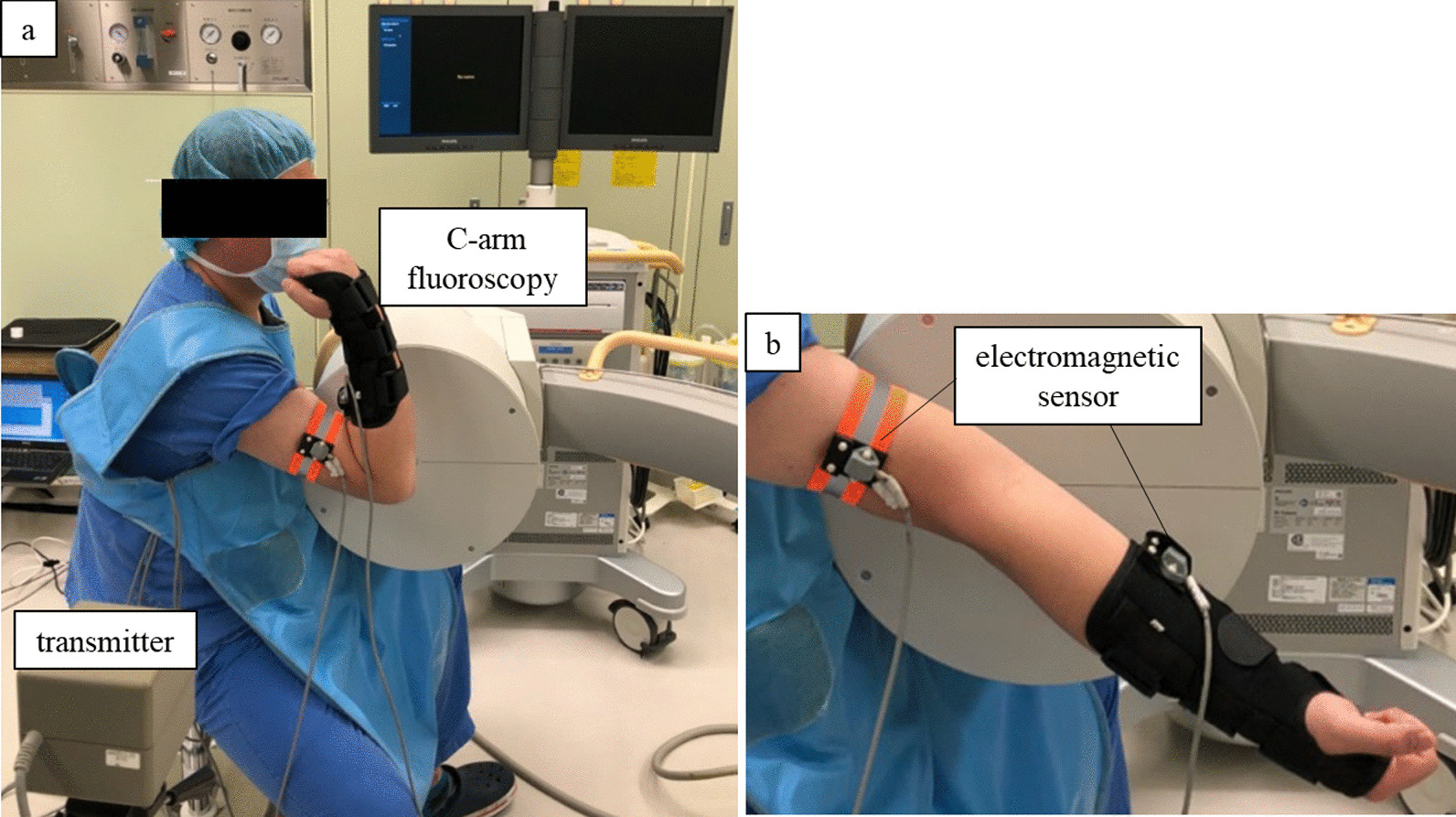
In vivo assessment of EMS. **a** Measurement at maximum elbow flexion. **b** Measurement at maximum elbow extension. The participants wore braces with sensors on their upper arms and forearms in a sitting position and performed active flexion and extension of the elbow joint with anterior elevation of the shoulder joint and forearm in maximal supination. EMS and radiological measurements were taken simultaneously

The protocol for in vivo measurements was reviewed and approved by our institutional review board (No. B210009). The study included six healthy men who volunteered to participate. The mean age was 33.4 ± 5.9 years (range, 24–41 years). The exclusion criteria included elbow pain, current elbow disorder, or a history of elbow surgery. The measurement brace with one sensor fixed was worn on the upper arm and forearm, as shown in Fig. 3. The third electromagnetic sensor was used to register the five landmarks in the same manner as the model bone. All participants were right-handed, and only the dominant side was evaluated. The participants were measured in a sitting position and performed active flexion and extension of the elbow joint with anterior elevation of the shoulder joint and forearm in maximal supination. Lateral radiographic images of the elbow joint were captured with the C-arm fluoroscopy system, BV Pulsera (Philips Medical Systems, The Netherlands) in the maximum flexion and extension positions for approximately 3 s. Radiographic images were considered a more accurate method for ROM measurement because of the advantage of being unaffected by the morphological features of the patient [6, 14, 16]. To obtain a complete lateral view of the elbow, the humeral capitellum and trochlea were photographed so that they appeared as concentric as possible [17]. Each participant was radiographed in maximal flexion and extension positions twice. In addition, once the brace and device were removed, this series of examinations was repeated twice. The total number of EMS and radiological measurements were 36 measurements in the flexion and extension positions of the elbow joint. To evaluate the accuracy and precision of the EMS measurement, the difference between the EMS and gold standard radiological measurements for each pair of 36 measurements was analyzed using the Bland–Altman method [18–20]. Reproducibility was assessed to calculate the intra-rater reliability and inter-rater reliability using ICCs.

### Statistical analysis

Statistical analyses were performed using SPSS software (PASW, version 18.0, SPSS Inc., Chicago, USA), and the data were expressed as the mean ± standard deviation. Statistical significance was set at *p* < 0.05. To evaluate the accuracy of EMS measurements and radiological measurements, the Bland–Altman method was used in this study. The Bland–Altman method is commonly used in method comparison studies, in which measurements are taken by two methods at the same time, and the differences between these measurements are examined [21].

## Results

### Assessment of EMS accuracy and reproducibility

**Fig. 4:**
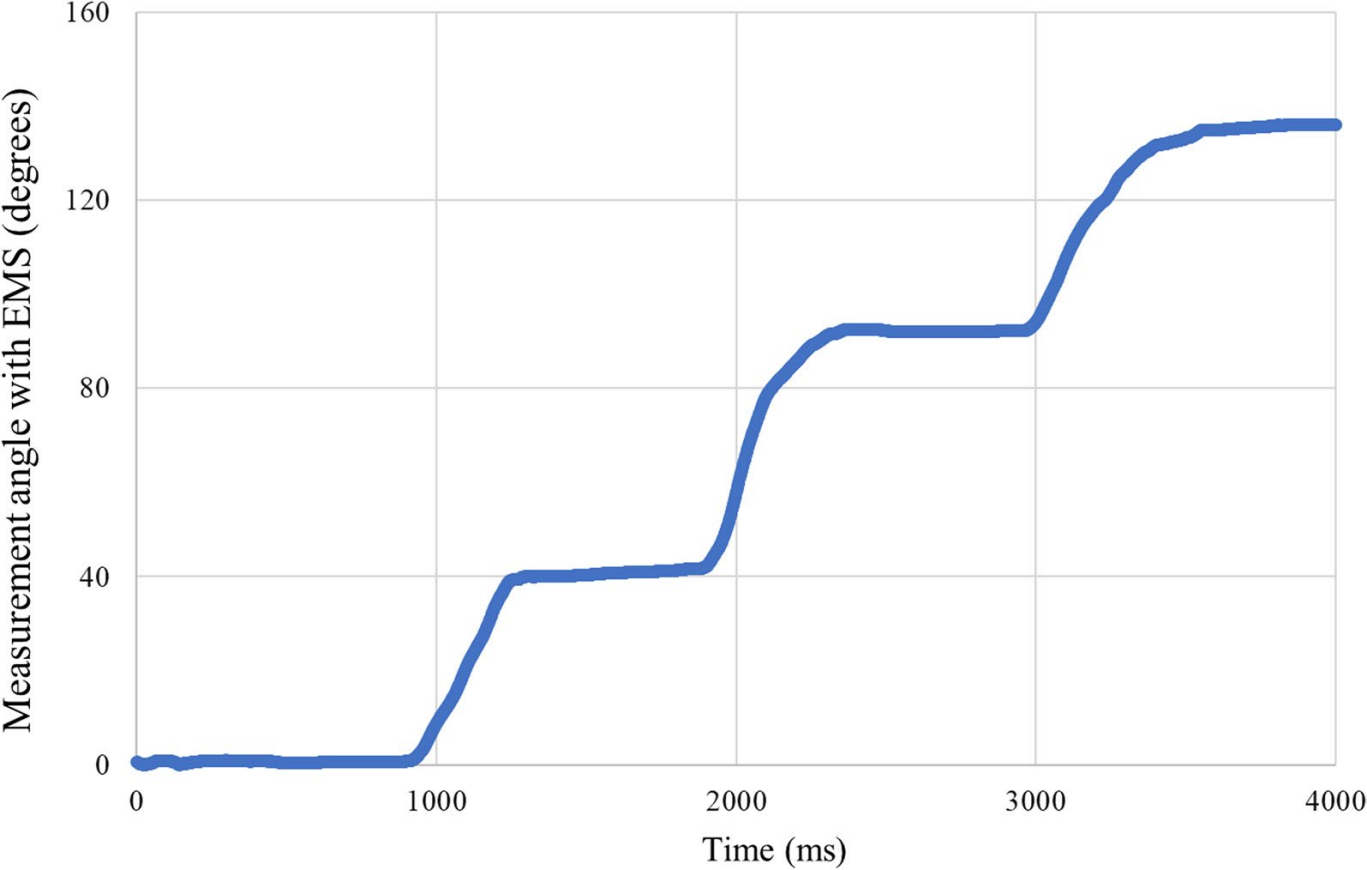
The measurement angle with EMS with a timeline. The timeline of the measurement angles with the EMS for one participant is shown in figure

The timeline of the measurement angles with the EMS for one participant is shown in Fig. 4. The measurement results are presented in Table 1. The mean flexion angles ± SD with EMS in the model bone elbow joint were 1.2° ± 2.0°, 45.4° ± 2.1°, 91.7° ± 2.4°, and 134.6° ± 2.7° in 0°, 45°, 90°, and 135° degrees of flexion with a goniometer, respectively. The error between the mean measurement angle with the EMS of the measurements and the actual measurement angle was less than 1.7° in all circumstances, and the SD was less than 2.7°. The Pearson's correlation coefficient was 0.999 (*p* < 0.0001).

**Table 1:** Measurement angle with EMS with a timeline

Actual measurement angle (°)	The mean measurement angle with EMS (°)	SD (°)
0	1.2	2.0
45	45.4	2.1
90	91.7	2.4
135	134.6	2.7

The intra-rater and inter-rater ICCs for EMS measurements are shown in Table 2. The ICC (1,3) indicating intra-rater reliability was 1.000, and the ICC (2,3) indicating inter-rater reliability was 1.000.

**Table 2:** Intra-rater and inter-rater ICCs for EMS measurements of model bone

	Intra-rater reliability		Inter-rater reliability	
	Mean ICC (1,10)	95% CI	Mean ICC (2,10)	95% CI
EMS measurement	1.000	0.999–1.000	1.000	0.998–1.000

### In vivo assessment of EMS

The mean angles of maximum extension and flexion measured with EMS were 4.7° ± 3.0° and 131.8° ± 13.0°, whereas the mean angles of the maximum extension and flexion measured with radiographic assessment were 2.7° ± 2.0° and 130.8° ± 4.5°, respectively. The measurement results are presented in Table 3. The mean angles of radiographic and EMS measurements in flexion and extension positions of the elbow joints and the Pearson correlation coefficient are shown. There were significant correlations between radiographic and EMS measurements in both the flexion and extension positions.

**Table 3:** Comparison of the difference and correlation between radiographic and EMS measurements

Range of motion	Radiographic measurement Mean ± SD (°)	EMS measurement Mean ± SD (°)	Pearson correlation coefficient between both methods (*p* value)
Extension	2.7 ± 2.0	4.7 ± 3.0	0.41 (*p* = 0.012)
Flexion	130.8 ± 4.5	131.8 ± 13.0	0.56 (*p* = 0.0004)

**Fig. 5:**
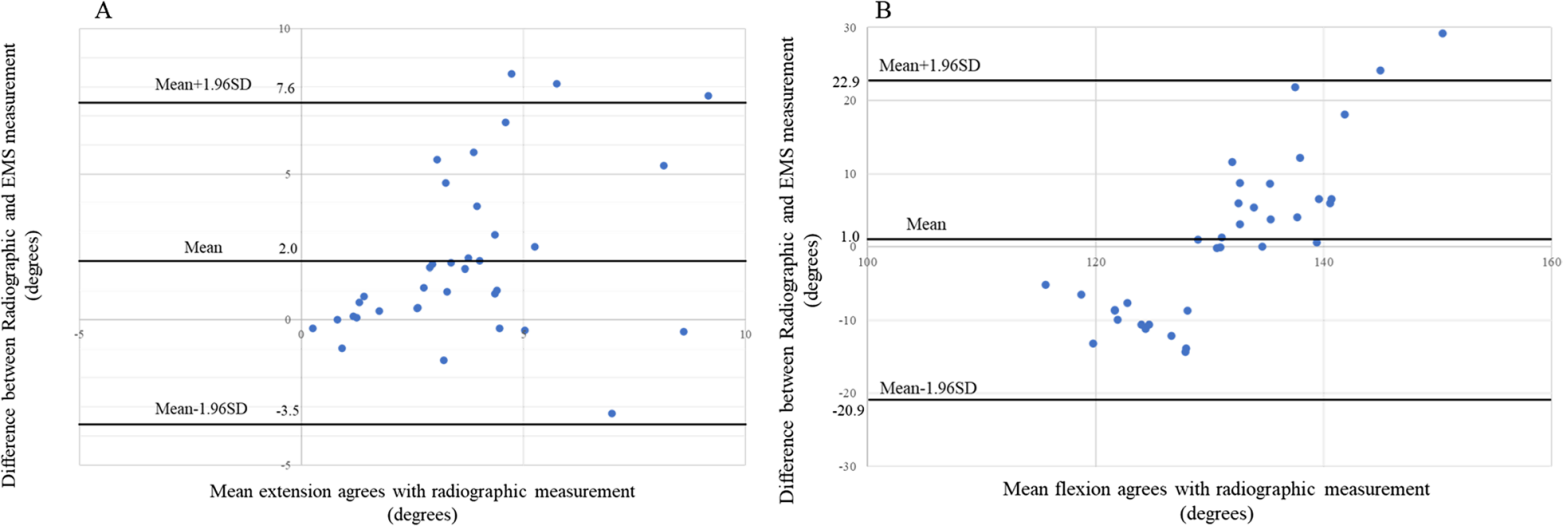
Bland–Altman plot.** A** Maximum extension of the elbow joint. **B** Maximal flexion of the elbow joint. Bland–Altman plots representing mean differences and 95% limits of agreement between the radiographic measurements and the EMS measurements of maximum elbow flexion and extension ROM. The middle line represents the mean difference, whereas the upper and lower lines indicate the 95% CI

The Bland–Altman analysis between the radiographic and EMS measurements in both flexion and extension positions is shown in Table 4. The CI reached ± 5.5° for the extension measurements; 95% of the EMS extension measurements were less than 5.6° different from the radiographic gold standard value. The CI reached ± 21.9° for the flexion measurements; 95% of the EMS flex measurements were less than 21.9° different from the radiographic gold standard value. The Bland–Altman plots are shown in Fig. 5. Zero points for both extension and flexion ROM measurements were included within the 95% CI of the difference between the radiographic and EMS measurements.

**Table 4:** Bland–Altman analysis of difference between radiographic and EMS measurements

Range of motion	Mean ± SD of difference (°)	Upper 95% CI limit (mean + 1.96SD)	Lower 95% CI limit (mean − 1.96SD)	Absolute maximal error (± 1.96SD)
Extension	2.0 ± 2.8	7.6	− 3.5	± 5.5
Flexion	1.0 ± 11.2	22.9	− 20.9	± 21.9

Intra-rater and inter-rater ICCs for radiographic and EMS measurements are shown in Table 5. The ICC (1,3) indicating intra-rater reliability for radiographic and EMS measurements was 1.000 and 0.998, respectively. The ICC (2,3) indicating inter-rater reliability for radiographic and EMS measurements was 0.999 and 0.998, respectively.

**Table 5:** Intra-rater and inter-rater ICCs for radiographic and EMS measurements of in vivo elbow joints

	Radiographic measurement	EMS measurement
*Intra-rater reliability*		
Mean ICC (1,3)	1.000	0.998
95% CI	0.999–1.000	0.995–0.999
*Inter-rater reliability*		
Mean ICC (2,3)	0.999	0.998
95% CI	0.997–1.000	0.993–0.999

## Discussion

Precise measurement of joint ROM is very important for treatment outcome assessments and reporting clinical research. The UG is a common clinical instrument used to evaluate the elbow joint and other major joints [1, 22, 23]. On the other hand, the reliability of the UG measurements is still under debate; the overall reliability of the UG measurements ranges from poor to excellent depending on the study. Fair to excellent reliability has been reported for the extension and flexion of elbow joints, with an intra-rater reliability of 0.45–0.99 and an inter-rater reliability of 0.53–0.98 [1, 6, 23–27]. Whether the examiner is an expert or a non-expert has some effect on the reliability of UG; Blonna et al. reported a lower inter-rater reliability of UG measurements in non-experts than in experts [28]. While radiographic measurements have the advantage of being very accurate due to the absence of soft tissue effects, they have the disadvantage of exposing the examinee to radiation [6, 16, 29]. In recent years, smartphone applications have been developed to measure ROM of the elbow joint; however, Vauclair et al. reported that the application measurements were not as accurate as the UG measurements and showed a tendency to overestimate the flexion angle measurement [30].

The EMS has shown high accuracy and reliability in the evaluation of knee instability associated with ACL injuries [7–13]. In this study, a new measurement method of ROM of the elbow joint using three-dimensional EMS was developed. The accuracy and reproducibility of the EMS measurement of elbow ROM were evaluated using a model bone that was not affected by the soft tissue. As a result of the accuracy assessment of the EMS measurements, the error between the mean measurement angle with the EMS and the actual measurement angle was less than 1.7° in all circumstances, the SD was less than 2.7°, and the Pearson's correlation coefficient was 0.999 (*p* < 0.0001), indicating high accuracy. Similarly, intra-rater and inter-rater reliabilities were calculated using the ICC for the reproducibility of the EMS measurements, and both ICC (1,10) and ICC (2,10) showed high reproducibility of 1.000. The values of the correlation coefficient were categorized according to this classification: slight; 0.00–0.20, fair; 0.21–0.40, moderate; 0.41–0.60, substantial; 0.61–0.80, and almost perfect; 0.81–1.00 [31].

Regarding the in vivo assessment, the EMS measurements for extension showed a similar SD to the radiographic measurements. On the other hand, the EMS measurements for flexion showed a larger SD of 13.0° compared to the radiographic measurements, resulting in increased variability. There were significant correlations between the EMS and radiographic measurements for both extension and flexion. In addition, the Bland–Altman analysis, which is currently the most commonly used method for comparative studies, was also used to evaluate the EMS and radiographic measurements [21]. In the current study, the 95% limit of agreement (LOA) was calculated as the mean difference ± 1.96 × SD according to Bland and Altman's method [32], and the 95% LOA Bland–Altman plots (Fig. 5) indicated that the two methods were almost in agreement for both extension and flexion. While the Bland–Altman plots for extension and flexion showed that there was no fixed error because the zero point was within the 95% LOA range, the flexion measurements showed the presence of proportional error, where the difference between the EMS and radiographic measurements increased as the measurement angle increased. This result could be supported by the increase in the SD of the EMS measurements for flexion compared with radiographic measurements. The results of the in vivo flexion measurements in the EMS measurements suggest that the sensors on the brace attached to the upper arm and forearm were slightly displaced by the contraction of the biceps during flexion. For the reproducibility of in vivo EMS measurements, intra-rater and inter-rater reliabilities were calculated using the ICC, and both the ICC (1,3) and ICC (2,3) showed a high reproducibility of 0.998. A previous report showed that the ICCs of the radiographic method ranged from 0.980 to 0.991 (6); the reliability of both methods in this study was also comparable.

There are no reports that have evaluated the ROM measurement of the elbow joint with EMS measurement and compared it to the radiographic measurement, which is the gold standard for accuracy. This new method of measuring the ROM of the elbow joint using the three-dimensional EMS showed quite high intra-rater and inter-rater reliabilities. This result suggests that EMS measurement has high accuracy, reliability, and reproducibility. Additionally, the advantages of this system are that it is non-invasive and that the sample rate of the EMS is 60 Hz, allowing for near-real-time measurements. The application of EMS to the elbow joint shown in this study and the real-time evaluation of EMS could allow further evaluation of acceleration, including evaluation of elbow joint instability in the future.

This study has several limitations. First, the effect of displacement of the sensors on the braces attached to the upper arm and forearm was not considered in this study. Considering that the validity of the SD is much less in the flexion measurement by EMS, the proportional error in flexion measurements shown by the 95% LOA Band-Altman plots between the radiographic and the EMS measurement may indicate soft tissue effects. Second, the sample size was small. The sample size for this study was calculated using G*Power (v 3.1; Universität Düseldorf) with a priori power analysis (*α* < 0.05, 1 − *β* ≥ 0.8) based on the similar previous study [30], showing *n* = 5 for flexion measurements and *n* = 7 for extension measurements. Considering this, it is suggested that the sample size number in this study is not extremely too small. Third, the subjects of this study were limited to healthy volunteers with no history of elbow joint disease. Therefore, this study did not evaluate the influence of elbow joint deformities resulting from traumatic fractures or epiphyseal line injuries on the accuracy of EMS. Although the elbow deformity is expected to affect the measurement of ROM not only in EMS in this study but also in UG and radiographic measurement, there is no report that evaluates it to the best of our knowledge and we would like to evaluate it in further studies in the future.

## Conclusions

This method of measuring the ROM of the elbow joint using the EMS showed high accuracy, reliability, and reproducibility. The current study results demonstrated the possibility of using an electromagnetic system to provide an accurate evaluation of the elbow joint in clinical settings.

## Data Availability

The datasets used and/or analyzed during the current study are available from the corresponding author on reasonable request.

## Abbreviations

ROM: Range of motion
EMS: Electromagnetic sensor system
ICC: Intraclass correlation coefficient
UG: Universal goniometer
LOA: Limit of agreement
